# Editorial

**DOI:** 10.34763/devperiodmed.20192304.245

**Published:** 2021-01-29

For many years the quarterly journal *Developmental Period Medicine/Medycyna Wieku Rozwojowego*, has been publishing articles on medical problems concerning pregnant women, newborns and children. True to this mission, the Editorial Committee wants to broaden the scope of the periodical to include medical, legal and ethical issues.

Pediatrics voices many medical concerns today. In a representative way it shows the contemporary paradox of medicine’s leaning on technology. A sufficient example is, for example, the problem of lethal disorders in newborns. Medicine has at its disposal a vast biotechnological potential – however, does "we can" always mean "we must"? Life brings forth many questions that medical personnel has to face on an almost daily basis: how to deal with suffering, which procedure to choose in each particular case, how to implement it? A multi-disciplinary outlook: medical, legal, and ethical will make it possible to approach the problems emerging in developmental period medicine in a deeper way. We also hope that rather than presenting only one point of view, it will provide a forum for a dialog among different fields of science, for example in connection with introducing many different technical and technological innovations. In this way *Developmental ^Period^ Medicine* is starting a new debate, introducing humanities to medical science. Such an approach is due to our awareness that the work of medical personnel today cannot be limited to being able to distribute pharmaceuticals or to correctly using complicated equipment. The physicians profession is not limited to carrying out a contract with the patient as a client. Every day, in many areas of clinical work, doctors have to make decisions of an ethical character, since their work is done in relation to another person – who is sufferng.

In the present issue, we feature the first two articles from the above cycle, which are devoted to the problems of palliative care in the perinatal period. Perinatology is a dynamically developing field, in which the advances in technology have recently made it possible to diagnose congenital disorders, including those of a lethal character, or genetic syndromes. The two papers presented two different approaches in palliative care. The first one used the example of the Interdisciplinary Team for Fetal Malformation at the Institute of Mother and Child in Warsaw, which has a history of 25 years. Three clinical situations were discussed in detail. The other article explained the functioning of the intrahospital perinatal hospice model in Wrocław. Its long experience has been recounted and the importance of this kind of institution existing under hospital conditions was emphasized. Both articles showed what looking after a pregnant woman is like when preparing for labor, in the course of labor and afterwards and how important psychological care is both for the pregnant woman and later – for the entire family.

The Editorial Committee invites doctors of different specialties, in particular obstetricians and gynecologists, oncologists, pediatricians, neurologists, surgeons, geneticists, psychologists, nurses and midwives as well as physiotherapists to contribute articles devoted to the above topics. We would also like to establish closer cooperation with philosophers, speciaists in ethics and lawyers, so they could coordinate articles devoted to the topics of the interconnections between medicine, law and ethics.

We hope that broadening philosophical and ethical knowledge and an exchange of ideas will help the whole medical community in its everyday clinical practice, in taking decisions, in initiating and holding discussions and in planning scientific work in this area.

*Magdalena Rutkowska, PhD with habilitation, Professor at the Institute of Mother and Child, Clinic of Neonatology and Intensive Therapy of Newborns*

